# Meaning-Centered Coping in the Era of COVID-19: Direct and Moderating Effects on Depression, Anxiety, and Stress

**DOI:** 10.3389/fpsyg.2021.648383

**Published:** 2021-03-17

**Authors:** Nikolett Eisenbeck, David F. Carreno, José Antonio Pérez-Escobar

**Affiliations:** ^1^Department of Psychology, University of Almería, Almería, Spain; ^2^Department of Personality, Evaluation and Psychological Treatment, Faculty of Psychology, University of Seville, Seville, Spain; ^3^Chair of History and Philosophy of Mathematical Sciences, Department of Humanities, Social and Political Sciences, ETH Zürich, Zurich, Switzerland

**Keywords:** COVID-19, meaning-centered coping, stress appraisal, psychological distress, depression, anxiety, existential positive psychology, positive psychology (PP1.0 and PP2.0)

## Abstract

The COVID-19 pandemic has subjected most of the world’s population to unprecedented situations, like national lockdowns, health hazards, social isolation and economic harm. Such a scenario calls for urgent measures not only to palliate it but also, to better cope with it. According to existential positive psychology, well-being does not simply represent a lack of stress and negative emotions but highlights their importance by incorporating an adaptive relationship with them. Thus, suffering can be mitigated (and transformed into growth) by, among other factors, adopting an attitude of positive reframing, maintaining hope, existential courage, life appreciation, engagement in meaningful activities, and prosociality. The conglomerate of these elements has been recently denominated as meaning-centered coping. In this study, we evaluated the protective role of this type of coping on mental health. A sample of 12,243 participants from 30 countries across all continents completed measures of Meaning-Centered Coping Scale (MCCS), depression, stress, anxiety and stressful COVID-19 related conditions they experienced. Results indicated that meaning-centered coping was strongly associated with diminished symptoms of stress, anxiety, and depression. Moreover, it moderated various relationships between vulnerability factors and markers of psychological distress, especially in the case of depression. These findings call for attention to meaning-centered coping approaches in the context of hardship, such as the current COVID-19 health crisis. In these difficult times, decision-makers and health organizations may integrate these approaches into their guidelines.

## Introduction

The COVID-19 pandemic has drastically affected people’s lives, negatively impacting multiple markers of physical and mental health (Cullen et al., 2020; Luo et al., 2020; Serafini et al., 2020; Sher, 2020). The conditions that produce these effects, such as enforced lockdowns and social isolation, are hardly avoidable or ameliorable. Focusing on the promotion of the already extended view that a very stressful situation like this leads to a proportional decrease in mental health is not sufficient. As such, an investigation of potential coping mechanisms against the specific conditions brought about by the pandemic would benefit millions of people.

In order to understand the significance and possible effects of stressful situations in psychology today, its virtues, its flaws and its exploits, we may look at the evolution of the concept of stress. At first, in psychology, a biomedical conceptualization of stress was adapted (e.g., Basowitz et al., 1955; Bliss et al., 1956; Tan and Yip, 2018), understanding it mainly as an unpleasant state of emotional and physiological arousal, carrying the assumptions that it is mostly harmful, and there is a somewhat linear relationship between the strain and the psychological response. This conceptualization carried inklings of linearity and negative value into psychology from its origins in physics and physiology (see for instance, how Selye’s findings in physiology led to the scientific legitimization of post-traumatic stress disorder in the DSM-III; Yehuda and McFarlane, 1995). General psychological distress can be divided into various facets, and oftentimes there is no terminological agreement among researchers. Although other definitions may be equally valid, this paper will adapt the terminology of Lovibond and Lovibond (1995) and Brown et al. (1997) which is fairly consistent with the tripartite model of anxiety and depression (Clark and Watson, 1991). That is, anxiety is defined by a physiological hyperarousal and subjective experience of negative affect, stress consists of the cognitive representation of said affect (tension, irritability, and getting easily frustrated) and depression is mostly but not exclusively characterized by a general absence of positive affect (dysphoria, hopelessness, etc.). It is indeed true, that, at least to some extent, stressful situations typically lead to increased stress, anxiety, and depression on the population level, as it could be observed during the COVID-19 pandemic (e.g., Salari et al., 2020).

However, we are far from assuming that stressors lead to psychological problems in all cases. It is well researched that protective and vulnerability/risk factors may make a person more or less prone to react with psychological distress to stressors (see for instance risk factors of depression: Dobson and Dozois, 2011). For example, during collective traumas, socially disadvantaged groups generally show worse psychological outcomes than their more advantaged counterparts (e.g., Reid, 2013). Several authors noted that mental health consequences due to the COVID-19 pandemic are more pronounced among vulnerable groups, such as females, younger people, people with low income levels, students, and those who are diagnosed with a mental health disorder or have a preexisting physical health condition (e.g., Alonzi et al., 2020; Asmundson et al., 2020; Fitzpatrick et al., 2020; Iob et al., 2020; Kajdy et al., 2020; Luo et al., 2020; Pieh et al., 2020; Purtle, 2020; Vahedian-Azimi et al., 2020; Xiong et al., 2020).

Beyond vulnerabilities and protective factors, we have a certain capability of transforming our psychological responses even in the most stressful situations, thus disrupting the linearity between stressors and our reactions to them. According to Lazarus (1993), some of the most relevant psychological aspects that define our reactions to stressors are coping (efforts to make a specific action related to the stressor) and appraisal (the person’s evaluation of the significance of the situation related to their well-being). Traditionally, coping mechanisms have been classified as problem or emotion-focused (see Folkman and Lazarus, 1984). Problem-focused coping strategies aim to resolve some aspect of the stressor, while emotion-focused strategies represent the attempt to regulate one’s emotions in the face of adversity. Data showed that during the COVID-19 pandemic, certain types of psychological coping mechanisms, especially problem-focused ones, were related to better mental health outcomes (e.g., Cai et al., 2020; Dawson and Golijani-Moghaddam, 2020; Huang et al., 2020).

Appraisal of stressful situations to optimize affective responses seems to be equally important (for a comprehensive review, see Jamieson et al., 2018). A stressor can be conceived as a challenge or as a threat, and depending on how it is appraised, it may have a very different effect on the individual and their coping strategy. Positive reappraisals and positive meanings influence the emotions the person experiences in the stressful encounter and can activate coping processes that support the positive affect domain (e.g., Folkman and Moskowitz, 2000). For instance, in a recent study, threat, challenge, centrality, and controllability appraisals regarding the COVID-19 health crisis were related to subjective well-being (Zacher and Rudolph, 2020).

Understanding this phenomenon can be useful to develop strategies to approach stressors also as challenges and opportunities for growth, instead of looking at them merely as threats to be ameliorated or avoided. For instance, in Chinese, “stress” is translated as “crisis,” which is written with the characters for “danger” and “opportunity.” This characterization in a way depicts the dialectic between harm and growth, which is in line with the conceptualization of psychological distress offered by existential positive psychology (Wong, 2009, 2011a) also known as PP2.0 (Wong, 2011b). Existential positive psychology not only focuses on the buffering effects of positive emotions and personal strengths in stressful situations (which are relevant contributions of positive psychology, see for instance Seligman and Csikszentmihalyi, 2000; Seligman, 2004), but also on the process of harnessing positive potentials from negative aspects of living, connecting the negative with the positive (e.g., Wong, 2011b; Kashdan and Biswas-Diener, 2014; Ivtzan et al., 2015). Indeed, the acceptance of negative affect seems to be necessary for healthy functioning (e.g., Levin et al., 2014).

The existential positive framework emphasizes meaning in life, virtue, resilience, and well-being as the pillars of personal flourishing. There is a vast literature showing a protective role of these processes. In particular, meaning in life seems to act as a buffer against psychological distress and foster resilience (e.g., Batthyany and Russo-Netzer, 2014; Gloria and Steinhardt, 2016). It also seems to play a transformative and rehabilitative role in disorders related to potentially traumatic events (Calhoun and Tedeschi, 2006; Khanna and Greyson, 2015) and substance abuse (Carreno and Pérez-Escobar, 2019). Especially, meaning in life has been shown to be negatively associated with depression both in cross-sectional studies (e.g., Steger et al., 2006; Carreno et al., 2020) and longitudinal ones (e.g., Disabato et al., 2017). According to systematic reviews and meta-analyses, interventions that foster meaning in life generally seem to reduce psychological distress and improve well-being (e.g., Vos, 2016; Vos and Vitali, 2018). These interventions seem to be especially effective among cancer patients, fostering improvements in spiritual well-being, quality of life, depression, and anxiety, among other areas (e.g., Breitbart et al., 2010; Chochinov et al., 2011). During the COVID-19 health crisis, a number of studies showed that higher levels of meaning in life were associated with lower levels of psychological distress (e.g., Arslan and Yildirim, 2020; Chao et al., 2020; Milman et al., 2020).

Based on Viktor E. Frankl’s work (1969,1985), Wong (1993, 2020a, b) proposed a coping style to face crises in a resilient and transformative way, involving strategies such as courage, responsibility, life appreciation, acceptance without judgment, positive reframing, self-transcendence, faith and hope (see also Wong et al., 2006). A recent study (Eisenbeck et al., unpublished) attempted to operationalize these ideas by creating a short scale that intends to encompass various elements of this approximation, denominated as the Meaning-Centered Coping Scale (MCCS). This instrument measures a set of attitudes and behaviors that include positive reframing of adversity, maintaining hope, existential courage, life appreciation, engagement in meaningful activities, and prosociality (see Eisenbeck et al., unpublished). Elements of this conceptualization of meaning-centered coping are well-studied, show strong relationships with both higher levels of well-being and decreased psychological distress (e.g., Nakamura and Csikzentmihalyi, 2003; Feldman and Snyder, 2005; Schueller and Seligman, 2010; Kleiman et al., 2013; Maddi, 2013; Van Tongeren et al., 2016; Klein, 2017; Jans-Beken and Wong, 2019) and are incorporated in the theory of PP2.0.

This coping style thus involves strategies that may prevent the development of psychopathological symptoms such as psychological stress, anxiety, and depression in situations such as the COVID-19 pandemic. Meaning-centered coping also intends to be “transformative,” as it deals with appraisal and coping processes that may help to maintain the positive affect domain even in the presence of negative affect, such as endowing stressful situations and emotions with a different, more positive meaning that can ultimately lead to growth (e.g., being grateful for parts of the experience) which is one of the central ideas of PP2.0. According to the tripartite model (Clark and Watson, 1991), anxiety, stress, and depression share the element of negative affect to some extent but only depression is characterized by low levels of positive affect. Based on its focus of transforming the negative to positive, it may be feasible to hypothesize that a meaning-centered coping style may be strongly correlated with depression (similarly to the sense of meaning in life which this coping style fosters, as it suggested by previous studies, see Steger et al., 2006). This hypothesized protective role against psychological distress and especially against depression may be extremely relevant during a global crisis like the COVID-19 pandemic, which entails a great deal of mostly unavoidable psychological hardship due to social isolation, confinement and fear of getting infected, among other factors.

In light of the aforementioned theoretical considerations, in the present study we analyzed the relationships between individual vulnerability factors, psychological distress and meaning-centered coping, hypothesizing that meaning-centered coping may play a role in this equation at various levels: directly affecting facets of psychological distress and by moderating the effect of vulnerability factors on psychological distress. We expect that these findings would be independent of the country of origin of the individuals.

Specifically, we postulate the following hypotheses:

H1. Certain demographic characteristics of the participants, such as gender, age, economic status and previous mental disorder diagnosis will be associated with increased levels of stress, anxiety, and depression (demographic vulnerability factors).

H2. Potentially harmful and more or less objectively measurable effects of the pandemic will also be related to markers of psychological distress, such as perceived lifestyle changes, economic and physical health effects of the pandemic, length of confinement and adherence to confinement (COVID-19 related vulnerability factors).

H3. Meaning-centered coping will be a negative predictor of all markers of psychopathology.

H4. The impact of the aforementioned risk factors on depression, anxiety and stress levels will be mitigated by higher levels of meaning-centered coping.

## Materials and Methods

### Participants

A total of 12,243 people from 30 countries participated in this study. Most of them were female and their average age was 35.44 years (SD = 13.24, range: 18–85). A detailed description of demographic variables can be observed in Table 1.

**TABLE 1:** Socio-demographic characteristics of the sample.

Country		Pandemic severity index	Gender (%)		Age		Education level (%)			Economic status (%)			Mental disorder diagnosis	≥21 days mostly at home
	*n*	1–3	F	M	*M* (SD)	Range	Secondary	University	Student	Above	Average	Below	Yes %	Yes %
Algeria	264	2	67.9	32.1	32.58 (10.35)	18–69	4.9	76.9	18.2	24.2	70.1	5.7	4.2	74.2
Argentina	163	1	74.8	25.2	37.50 (11.67)	18–70	16.6	70.6	12.9	8.6	70.6	20.9	11.0	8.0
Australia	63	2	85.7	14.3	44.19 (10.65)	18–73	7.9	87.3	4.8	50.8	46.0	3.2	15.9	55.6
Bangladesh	344	1	39.8	60.2	25.35 (7.41)	18–78	9.9	43.3	46.8	7.6	86.0	6.4	0	2.0
Brazil	386	2	75.6	24.4	37.94 (12.71)	18–77	8.8	81.1	10.1	31.1	59.6	9.3	22.8	86.8
Canada	394	2	52.4	47.6	36.80 (13.23)	18–84	15.2	74.6	10.2	24.9	61.7	13.5	15.7	73.1
Colombia	130	2	88.5	11.5	39.54 (12.27)	18–70	15.4	69.2	15.4	7.7	73.1	19.2	11.5	4.6
Egypt	293	1	71.0	29.0	37.23 (11.50)	18–84	13.0	81.6	5.5	34.1	58.0	7.8	2.7	46.1
France	481	3	77.3	22.7	43.69 (11.51)	18–81	10.4	83.6	6.0	20.2	69.2	10.6	3.3	86.3
Germany	296	3	69.3	30.7	40.78 (15.04)	18–79	32.1	58.8	9.1	40.5	49.7	9.8	5.1	75.0
Hungary	282	2	89.3	10.7	37.36 (12.40)	18–71	22.3	70.2	7.4	26.2	63.8	9.9	7.1	6.7
India	602	1	56.7	43.3	25.75 (7.94)	18–85	7.6	56.3	36.0	11.8	81.6	6.6	1.0	24.1
Indonesia	289	1	73.0	27.0	24.78 (9.46)	18–59	9.3	33.2	57.4	9.7	84.1	6.2	4.2	54.3
Italy	536	3	75.9	24.1	34.50 (14.67)	18–80	37.1	46.5	16.4	8.2	77.1	14.7	4.7	50.6
Lebanon	329	2	65.3	34.0	28.34 (11.59)	18–69	7.0	57.4	35.6	19.8	73.3	7.0	10.6	98.2
Mexico	717	2	80.3	19.7	40.76 (13.34)	18–80	4.5	91.8	3.8	43.1	16.6	40.3	9.6	77.5
New Zealand	73	2	80.8	19.2	44.89 (11.30)	20–74	23.3	76.7	0	43.8	49.3	6.8	41.1	56.2
Nigeria	435	1	31.5	68.5	33.34 (8.67)	19–64	0.7	87.8	11.5	7.1	79.3	13.6	0	13.3
Pakistan	426	1	61.3	38.7	28.59 (10.33)	18–80	8.9	59.6	31.5	30.8	64.3	4.9	1.4	79.8
Poland	332	2	81.6	18.4	32.69 (12.18)	18–82	10.2	67.2	22.6	26.5	68.7	4.8	17.2	73.5
Portugal	522	3	72.4	27.6	38.93 (12.20)	18–75	21.1	74.1	4.8	10.3	80.8	8.8	6.9	91.8
Romania	557	2	70.7	29.3	32.78 (11.59)	18–69	12.9	69.7	17.4	16.2	78.5	5.4	2.0	1.8
Russia	324	2	89.8	10.2	44.38 (11.03)	19–79	2.5	93.5	4.0	14.2	75.6	10.2	5.2	87.7
Slovenia	1345	2	83.2	16.8	34.39 (13–67)	18–81	27.9	54.6	17.5	11.9	78.2	9.9	5.5	33.8
Spain	723	3	77.0	23.0	36.51 (11.81)	18–73	14.9	72.6	12.4	7.9	75.9	16.2	11.5	1.1
Sweden	314	3	84.3	15.7	41.05 (12.23)	20–75	14.6	73.6	11.8	40.1	51.9	8.0	11.5	89.2
Thailand	422	1	35.1	64.9	34.23 (10.83)	18–70	3.1	86.7	10.2	14.9	73.9	11.1	4.0	69.9
Turkey	322	2	60.9	39.1	27.27 (8.59)	18–61	4.7	62.4	32.9	12.4	70.8	16.8	6.2	51.2
United Kingdom	514	3	88.1	11.9	42.33 (15.22)	18–76	28.8	48.8	22.4	17.9	62.1	20.0	25.7	57.8
United States	365	3	78.6	21.4	44.37 (12.31)	18–77	14.2	56.7	29.0	29.0	52.3	18.6	23.0	65.5
Total	12243	–	71.0	28.8	35.55 (13.21)	18–85	14.7	67.6	17.7	19.5	68.0	12.4	8.3	51.6

### Measures

#### Depression, Stress, and Anxiety

The Depression Anxiety and Stress Scale (DASS-21; Henry and Crawford, 2005), which is the short version of the original, 42-item DASS scale (see Brown et al., 1997) was used in this study. The questionnaire contains 21 items with three subscales measuring depression, anxiety and stress (7 items each). Respondents have to rate each item on a scale of 0 (did not apply to me at all) to 3 (applied to me very much, or most of the time). As raw scores are typically multiplied, total scores are 42 on each subscale and 126 on the total scale (referred to as total psychological distress). Higher scores indicate higher levels of psychological problems. The measure showed good psychometric properties in the past (e.g., Henry and Crawford, 2005). Alphas in the present sample ranged between 0.83 and 0.94.

#### Meaning-Centered Coping

The MCCS (Eisenbeck et al., unpublished) measures a coping strategy based on the creation of personal meaning, as proposed by existential positive psychology. The measure includes 9 items about life appreciation (e.g., “I am grateful for my life as it is.”) maintaining hope, positive reframing, courage (e.g., “I will get out of this situation stronger than I was before.”) and involvement in meaningful and prosocial activities (e.g., “I help others during this time.”). Items are rated on a 7-point Likert scale from (I do not agree at all) to 7 (I completely agree). Validation of the questionnaire showed a stable one-factor solution, good internal consistency, convergent and divergent validity, and test–retest data in all languages used in the present study (Eisenbeck et al., unpublished). In the present sample, Cronbach’s alphas were between 0.81 and 0.95 in each country (total sample: α = 0.88).

#### Possible Risk Factors

On one hand, demographic data (age, gender, economic status, education level, and the existence of previous mental health disorder diagnosis) that can pose a risk factor for depression, stress, and anxiety during the COVID-19 health crisis were collected. On the other hand, self-reported data about variables that more or less can be reliably assessed and reported by the participants regarding the intensity of some of the effects of the pandemic were collected. The following variables were measured: (a) economic impact (“This pandemic affects me economically for the worse.”); (b) physical health effects (“This pandemic negatively affects my physical health, although not infected.”); (c) general lifestyle changes (“This pandemic has caused me significant lifestyle changes.”); (d) adherence to confinement rules (“I stay at home except for strictly necessary things (e.g., to go to work, get food or medicine); and (e) duration of confinement (“If you have had to stay at home due to the pandemic, how many days has it been since you started staying home?”). Items were measured on Likert scales from 1 (I do not agree at all) to 7 (I completely agree) with the exception of the last one.

In addition, country-level data that may be related to the psychological distress levels of people during the COVID-19 pandemic were included in the study: (a) objective severity index of the pandemic during data collection in each country: countries with fewer than 100 reported infections per million inhabitants were assigned a severity level of 1, countries with between 100 and 300 infections per million inhabitants a severity level of 2, and countries with more than 2000 cases per million inhabitants a severity level of 3 (data obtained from worldmeters.info); and (b) GDP of each participating country (source: worldbank.org).

### Procedure

The present research was part of a larger international project assessing the psychological effects of the COVID-19 pandemic between March and June 2020. Investigators were recruited through ResearchGate announcements. Interested researchers were included in the project if they were willing to adapt the questionnaire package to their languages and to recruit approximately 250 or more participants in their respective countries. All adaptations were realized according to the best practices (Beaton et al., 2000). The researchers recruited the study participants through direct e-mail invitations (i.e., they sent emails to their professional and personal contacts) and social media announcements (Facebook and Instagram). No incentives were given to the participants, with the exception of part of the Canadian sample that was recruited via MTurk. The participation was voluntary and anonymous. Nevertheless, they were given the option to provide their e-mail addresses if they wished to participate in similar studies in the future. This information was handled separately from their other responses in order to maintain anonymity. Respondents filled in the survey (demographic data, risk factors, the DASS-21 and the MCCS) in approximately 10 min. At the end of the participation, they were fully debriefed.

### Data Analysis

Data were tested with SPSS (Version 25). Prior to the analysis, data were tested for normality and missing values. Data were removed for respondents who left at least 50% of any given questionnaire unfinished or showed straight-lining. People with COVID-19 diagnosis were less than 1% of the dataset, and they were excluded. Missing data in the formal questionnaires was less than 0.1%, (missing completely at random) and were replaced with the expectation-maximization procedure. Missing data on demographic variables the duration of confinement was 0.21%. It was mostly due to technical error, as age was not measured in 136 cases in the US sample. Because of the low percentage of demographic missing data, they were replaced with the country average. Duration of the confinement was dichotomized to represent values below and above the median of the sample (*Mdn* = 21 days). Possible outliers were not removed from the database. Data from different countries were compared with analyses of variance (continuous variables) and with Cramer’s *V* (dichotomous variables).

Four separate analyses of multilevel modeling (MLM) on depression, anxiety, stress, and the total DASS-21 scores were conducted to assess the effects of individual and country-level variables and the moderating role of the MCCS, using the restricted maximum likelihood method. Education status was dichotomized for this analysis to assess possible effects of student status on mental health. First, baseline models with random intercepts were tested to determine whether MLM was warranted. Models were compared to the full, nested models by χ^2^ difference tests using the maximum likelihood estimation method. The moderator role of the MCCS between the predictor and outcome variables was further assessed with simple slopes at different levels of the moderator variable: at low (−1 SD: 9–35) points), mean (0 SD: 36–58 points), and high levels (+1 SD: 59–63 points) using the PROCESS macro (Hayes, 2017). Simple slopes were computed in all cases, even when the main interaction term was not significant to assess possible trends. For easier interpretation and comparability, the use of standardized variables was maintained throughout the analyses.

## Results

As expected, samples from different country of origin significantly differed on almost all demographic variables, such as gender, Cramer’s *V* = 0.330, *p* < 0.001, age, *F*(29) = 89.07, *p* < 0.001, $\eta_{\text{p}}^{2}$ = 0.175, economic status, *F*(29) = 19.05, *p* < 0.001, $\eta_{\text{p}}^{2}$ = 0.043, education level *F*(29) = 39.04, *p* < 0.001, $\eta_{\text{p}}^{2}$ = 0.085, mental disorder diagnosis, Cramer’s *V* = 0.265, *p* < 0.001, percentage of students, Cramer’s *V* = 0.325, *p* < 0.001, and duration of the confinement, Cramer’s *V* = 0.625, *p* < 0.001. Differences were also observed on depression, *F*(29) = 27.07, *p* < 0.001, $\eta_{\text{p}}^{2}$ = 0.062, anxiety, *F*(29) = 25.71, *p* < 0.001, $\eta_{\text{p}}^{2}$ = 0.058, stress, *F*(29) = 31.05, *p* < 0.001, $\eta_{\text{p}}^{2}$ = 0.069, the DASS-21 total scores, *F*(29) = 28.42, *p* < 0.001, $\eta_{\text{p}}^{2}$ = 0.063, and the MCCS, *F*(29) = 26.00, *p* < 0.001, $\eta_{\text{p}}^{2}$ = 0.058, These results warrant the inclusion of the variable country into all analyses of the study. Detailed data of the variables measured in each country can be observed in Tables 1, 2.

**TABLE 2:** Average data of the measures of the study in each country.

	DASS depression	DASS anxiety	DASS stress	DASS-21 total	Meaning-centered coping	Economic impact	Physical health effects	Lifestyle changes	Adherence to confinement
	*M* (SD)	*M* (SD)	*M* (SD)	*M* (SD)	*M* (SD)	*M* (SD)	*M* (SD)	*M* (SD)	*M* (SD)
Algeria	9.74 (9.70)	8.20 (9.00)	11.99 (10.63)	29.94 (27.25)	48.64 (11.92)	4.86 (2.36)	3.62 (2.34)	4.20 (2.17)	6.19 (1.65)
Argentina	8.88 (9.47)	7.45 (8.35)	12.52 (10.01)	28.85 (25.27)	49.21 (11.71)	5.15 (2.03)	3.95 (2.13)	5.23 (1.96)	6.33 (1.47)
Australia	8.19 (8.13)	5.46 (6.39)	13.56 (9.65)	27.21 (20.87)	50.27 (10.90)	4.33 (2.09)	3.33)1.94)	5.81 (1.55)	6.10 (1.43)
Bangladesh	13.30 (9.24)	12.63 (9.14)	15.01 (9.61)	40.94 (25.87)	39.17 (13.18)	4.73 (2.19)	4.15 (2.18)	4.35 (2.00)	5.07 (2.01)
Brazil	12.13 (10.47)	6.61 (7.68)	12.89 (10.21)	31.63 (25.76)	44.63 (12.42)	4.30 (2.07)	4.28 (1.91)	5.49 (1.56)	6.62 (0.90)
Canada	11.36 (10.34)	6.75 (7.61)	13.18 (9.95)	31.28 (25.02)	46.35 (10.75)	4.10 (1.96)	4.02 (1.90)	5.64 (1.40)	6.19 (1.20)
Colombia	10.92 (10.50)	8.38 (9.89)	15.58 (11.12)	34.89 (28.46)	46.21 (12.37)	4.46 (2.07)	3.75 (2.16)	5.24 (1.83)	6.30 (1.49)
Egypt	10.62 (9.68)	7.45 (8.13)	12.31 (10.03)	30.38 (25.18)	49.92 (11.87)	3.08 (1.73)	4.62 (1.50)	4.73 (1.52)	4.92 (1.23)
France	10.00 (9.84)	5.21 (6.96)	12.81 (10.67)	28.02 (24.10)	43.82 (10.24)	3.12 (2.07)	2.92 (1.81)	4.59 (1.86)	6.27 (1.35)
Germany	5.99 (7.73)	2.71 (5.02)	8.78 (8.86)	17.48 (18.67)	46.09 (9.34)	2.98 (1.98)	2.90 (1.86)	5.03 (1.80)	5.38 (1.67)
Hungary	9.22 (9.73)	7.10 (7.95)	15.03 (9.89)	31.35 (23.83)	45.41 (10.62)	4.18 (1.91)	3.40 (1.94)	5.48 (1.67)	6.45 (1.17)
India	6.41 (7.53)	6.19 (7.06)	6.90 (7.60)	19.50 (20.41)	46.73 (12.88)	3.54 (2.04)	2.90 (2.02)	4.38 (2.10)	5.88 (1.79)
Indonesia	7.74 (7.76)	7.47 (7.40)	12.53 (9.18)	27.74 (22.26)	53.69 (7.89)	4.51 (1.81)	3.35 (1.90)	4.94 (1.79)	6.04 (1.53)
Italy	10.98 (7.91)	5.62 (6.34)	13.24 (8.31)	29.84 (19.20)	44.86 (10.07)	4.13 (2.08)	3.51 (1.83)	5.37 (1.78)	6.58 (1.10)
Lebanon	13.62 (11.32)	9.57 (9.55)	15.74 (10.54)	38.93 (28.32)	46.40 (12.07)	5.00 (1.80)	3.97 (2.13)	5.38 (1.61)	5.97 (1.45)
Mexico	8.26 (9.01)	6.60 (8.28)	13.55 (9.82)	28.42 (24.72)	51.98 (10.42)	4.72 (1.98)	3.43 (2.15)	5.62 (1.79)	6.27 (1.34)
New Zealand	11.51 (10.55)	7.34 (9.01)	15.23 (9.66)	34.08 (25.49)	46.93 (11.43)	4.33 (1.99)	3.33 (2.05)	5.62 (1.67)	6.25 (1.21)
Nigeria	5.53 (7.49)	5.00 (7.24)	6.57 (8.04)	17.10 (21.23)	48.18 (13.08)	4.53 (2.08)	3.07 (2.02)	4.41 (2.11)	5.31 (2.06)
Pakistan	11.54 (9.31)	10.02 (8.79)	13.37 (9.22)	34.93 (25.53)	42.42 (14.20)	4.05 (2.11)	3.34 (2.03)	4.53 (1.94)	5.34 (2.03)
Poland	12.59 (11.42)	8.90 (9.45)	17.36 (11.08)	38.85 (28.83)	43.05 (11.79)	4.44 (2.14)	4.19 (1.97)	5.78 (1.50)	6.28 (1.31)
Portugal	7.49 (7.49)	5.26 (7.20)	13.36 (9.88)	26.11 (22.06)	46.72 (9.85)	4.30 (2.07)	3.72 (1.91)	5.28 (1.75)	6.31 (1.39)
Romania	8.30 (8.54)	6.02 (7.73)	11.16 (9.66)	25.48 (23.16)	47.78 (10.55)	4.55 (1.98)	3.11 (1.82)	4.22 (1.84)	6.01 (1.72)
Russia	9.19 (8.41)	6.23 (6.96)	14.17 (9.62)	29.59 (21.72)	48.66 (10.03)	4.25 (2.11)	3.92 (2.01)	4.53 (1.91)	5.95 (1.59)
Slovenia	10.39 (10.34)	5.85 (7.60)	14.08 (10.74)	30.33 (25.96)	48.21 (9.76)	4.06 (2.03)	3.59 (1.99)	5.32 (1.69)	6.36 (1.23)
Spain	9.04 (9.51)	6.47 (7.66)	13.04 (9.29)	28.55 (23.79)	45.52 (12.45)	4.51 (2.18)	3.95 (2.02)	5.18 (1.86)	6.35 (1.39)
Sweden	9.60 (8.61)	3.79 (6.46)	11.34 (8.57)	24.73 (19.61)	45.08 (9.72)	2.85 (2.04)	3.03 (1.96)	4.24 (1.86)	5.03 (1.77)
Thailand	5.54 (7.17)	4.92 (6.24)	8.96 (8.17)	19.42 (20.20)	44.74 (10.85)	3.89 (1.97)	3.51 (1.76)	4.97 (1.93)	5.38 (2.09)
Turkey	12.97 (10–36)	8.55 (8.00)	14.35 (10.23)	35.87 (25.86)	47.00 (10.93)	3.64 (2.16)	3.85 (2.16)	5.21 (1.87)	6.30 (1.41)
United Kingdom	15.06 (11.80)	10.66 (11.04)	18.42 (11.75)	44.14 (31.58)	43.80 (12.11)	3.61 (2.16)	3.80 (2.05)	5.19 (1.90)	6.14 (1.53)
United States	11.59 (11.08)	6.82 (8.13)	13.94 (10.98)	32.35 (27.21)	48.47 (10.88)	3.99 (2.08)	3.77 (2.04)	5.40 (1.66)	6.24 (1.32)
Total	9.82 (9.71)	6.80 (8.08)	12.90 (10.15)	29.52 (25.19)	46.64 (11.53)	4.12 (2.12)	3.59 (2.02)	5.03 (1.87)	6.04 (1.58)

### Predictors of Psychopathology

#### Main Predictors

Effect of country was significant in all baseline models: depression (−2LL = 34092.194; Wald *Z* = 3.65, *p* < 0.001, ICC = 0.062), anxiety (−2LL = 34146272; Wald *Z* = 3.64, *p* < 0.001, ICC = 0.062), stress (−2LL = 34002.516; Wald *Z* = 3.66, *p* < 0.001, ICC = 0.066), and the DASS-21 total scores (−2LL = 34073.175; Wald *Z* = 3.66, *p* < 0.001, ICC = 0.064). Although these data suggest that country-level variables explained a small part of the variance, as it was significant, models including both individual and country-level variables were tested. According to the hypotheses, interaction terms were included (see Table 3).

**TABLE 3:** Four final multilevel models predicting markers of psychological distress.

	1. Depression	2. Anxiety	3. Stress	4. DASS-21 total
	*B* (SE)	*B* (SE)	*B* (SE)	*B* (SE)
***Individual-level variables***				
Gender (female/male)	0.175 (0.017)***	0.207 (0.019)***	0.288 (0.018)***	0.250 (0.018)** *
Age	−0.121 (0.009)***	−0.101 (0.009)***	−0.144 (0.009)***	−0.137 (0.009)***
Student status (yes/no)	−0.077 (0.008)**	−0.065 (0.024)**	−0.021 (0.008)	−0.059 (0.008)**
Economic status	0.038 (0.008)***	0.046 (0.008)***	0.006 (0.008)	0.031 (0.008)***
Mental disorder diagnosis	−0.522 (0.029)***	−0.642 (0.031)***	−0.488 (0.030)***	−0.604 (0.029)***
≥ 21 days mostly at home	0.036 (0.019)*	0.004 (0.020)	0.015 (0.002)	−0.019 (0.019)
MCCS	−0.314 (0.008)***	−0.128 (0.009)***	−0.177 (0.009)***	−0.234 (0.009)***
Economic impact	0.057 (0.008)***	0.023 (0.009)**	0.038 (0.009)***	0.045 (0.008)***
Physical health effects	0.220 (0.008)***	0.234 (0.009)***	0.252 (0.009)***	0.261 (0.008)***
Lifestyle changes	0.106 (0.009)***	0.071 (0.009)***	0.146 (0.009)***	0.123 (0.009)***
Adherence to confinement	−0.049 (0.009)***	−0.058 (0.010)***	−0.053 (0.009)***	−0.059 (0.009)***
***Country-level variables***				
Severity of the pandemic	0.012 (0.050)	−0.055 (0.051)	0.077 (0.052)	0.018 (0.051)
GDP	0.019 (0.046)	−0.049 (0.046)	−0.001 (0.047)	−0.012 (0.046)
***Interactions***				
Gender ^X^ MCCS	0.037 (0.008)***	0.022 (0.008)**	0.023 (0.008)**	0.030 (0.008)***
Age ^X^ MCCS	0.021 (0.008)**	0.003 (0.009)	−0.004 (0.008)	0.005 (0.008)
Student status ^X^ MCCS	0.001 (0.007)	0.005 (0.009)	0.001 (0.008)	−0.001 (0.008)
Economic status ^X^ MCCS	−0.034 (0.007)***	−0.036 (0.008)***	−0.019 (0.008)*	−0.032 (0.007)***
Mental disorder diagnosis ^X^ MCCS	−0.030 (0.007)***	−0.008 (0.007)	0.003 (0.007)	−0.013 (0.007)
≥ 21 days mostly at home ^X^ MCCS	−0.019 (0.008)*	−0.001 (0.008)	−0.004 (0.008)	−0.009 (0.008)
Economic impact ^X^ MCCS	−0.019 (0.008)*	−0.013 (0.009)	−0.006 (0.009)	−0.014 (0.009)
Physical health effects ^X^ MCCS	−0.030 (0.009)***	−0.023 (0.009)*	−0.017 (0.009)*	−0.026 (0.009)**
Lifestyle changes ^X^ MCCS	−0.036 (0.009)***	−0.012 (0.009)	−0.023 (0.009)*	−0.027 (0.009)**
Adherence to confinement ^X^ MCCS	−0.020 (0.008)**	0.010 (0.008)	−0.003 (0.008)	−0.005 (0.008)
***Covariance parameters***				
Residual variance	0.670 (0.009)***	0.780 (0.009)***	0.720 (0.009)***	0.681 (0.009)***
Intercept variance	0.027 (0.008)**	0.029 (0.009)**	0.030 (0.009)**	0.029 (0.008)**
-2 log likelihood	30072.723	31917.471	30956.135	30260.236
χ^2^ (*df*) difference with null model (ML)	4187.424 (23)***	2393.379 (23)***	3212.608 (23)***	3980.469 (23)***

*p < 0.05; **p < 0.01; ***p < 0.001. All coefficients are standardized. Each column represents a separate model. MCCS = Meaning-Centered Coping scale.

Results indicated that gender, age, mental disorder diagnosis, meaning-centered coping, perceived economic impact, physical health effect and lifestyle changes due to the pandemic, together with adherence to confinement, had a significant impact on all markers of psychological distress (depression, anxiety, stress, and their combined score). Specifically, females, older participants, participants with mental disorder diagnosis and people who reported having lower levels of meaning-centered coping, higher levels of economic and physical health impact, significant lifestyle changes, and lower levels of adherence to confinement reported higher levels of negative emotional states.

Being a student and having lower economic status were related to increased depression, anxiety and the total DASS-21 scores but not to higher stress levels. More than 21 days in confinement predicted depression levels but not stress or anxiety levels. Country-level variables had no effect on the dependent variables of any of the four models.

### The Moderating Role of Meaning Centered Coping

Moderating effects of meaning-centered coping levels, as measured by the MCCS, between individual-level variables and markers of psychological distress were tested in all four models (see Tables 3, 4). Results showed that the meaning-centered coping moderated the effects of all tested variables on depression with the exception of student status: gender, age, economic status, psychological diagnosis, 21 or more days spent at home, economic and physical health impact of the pandemic and perceived lifestyle changes. Meaning-centered coping also moderated the effects of gender, economic status and physical health impact of the pandemic on anxiety. In case of stress and the total DASS-21 scores, it had a moderating role in the effect of gender, economic status, physical health impact and perceived lifestyle changes due to the pandemic. In all significant interactions, the same pattern was observed: higher levels of the MCCS were related to diminished relationships between the aforementioned predictors and markers of psychological distress (see Table 4). That is, meaning-centered coping acted as a buffer between certain vulnerability factors and depression, anxiety, stress, and their total scores.

**TABLE 4:**
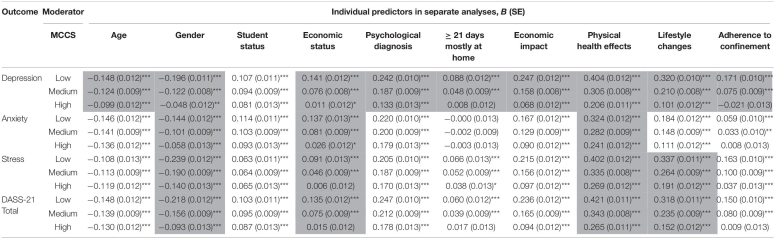
Moderating role of Meaning-centered coping: results of 36 separate analyses.

*p < 0.05; **p < 0.01; ***p < 0.001. Significance refers to the individual slopes, while significant interactions according to the original full models are highlighted. All variables were standardized. MCCS = Meaning-Centered Coping Scale.

## Discussion

The purpose of this study was to evaluate the relationships between several risk factors and markers of psychological distress during the initial phases of the COVID-19 pandemic, as well as to assess the protective role of meaning-centered-coping in their relations. Four hypotheses were tested in a sample of 12,234 in 30 countries from all continents.

In line with H1, the study confirmed that there are demographic vulnerability factors of psychological distress during the COVID-19 health crisis over which the individual has little to no control, at least in the short term. This is the case of age, gender, economic status, and being a student. These findings are congruent with recent studies showing that females, younger people, students, and those with lower economic status may be the most vulnerable to developing psychological problems during the COVID-19 pandemic (e.g., Alonzi et al., 2020; Asmundson et al., 2020; Fitzpatrick et al., 2020; Iob et al., 2020; Kajdy et al., 2020; Luo et al., 2020; Pieh et al., 2020; Purtle, 2020; Vahedian-Azimi et al., 2020; Xiong et al., 2020). The results also demonstrated that people with mental disorder diagnosis showed higher levels of all aspects of emotional disturbance than their non-diagnosed counterparts. In fact, this condition was the most prominent risk factor among all assessed individual predictors (for similar results, see Asmundson et al., 2020).

Other important vulnerability factors linked to increased distress on an individual basis were perceived economic impact, physical health effects, and lifestyle changes due to the COVID-19 pandemic, as well as lack of adherence to confinement (H2). Among the COVID-19-related factors, noxious effects of the pandemic on physical health (unrelated to the actual illness) was the strongest predictor of all negative emotional states. As previous studies have reported, people with a preexisting physical condition can be extremely vulnerable to the effects of the pandemic (see for instance, Alonzi et al., 2020). Individual duration of confinement was only related to depression in our sample. The severity of the pandemic in each country (percentage of confirmed COVID-19 cases during the data collection) and GDP did not directly predict any facet of psychological distress. These findings indicate that both demographic vulnerability markers and effects of the pandemic at the level of individuals (lifestyle change, economic, and physical problems) were generally related to declined mental health during the COVID-19 crisis, independently from the country of origin of the participants.

However, this alone may lead to an overly simplified picture. As predicted by H3, meaning-centered coping was associated with decreased levels of stress, anxiety and depression, suggesting that this coping style is related to the diminished experience of these negative emotional states, especially depression. H4 was formulated to support its buffer effects, expecting that meaning-centered coping would moderate the relationship between risk factors (both demographic ones and those directly related to the pandemic) and aspects of psychological distress. Results confirmed this hypothesis, showing that meaning-centered coping had a moderating effect of all aforementioned significant demographic and pandemic-related individual vulnerability factors (age, gender, mental disorder diagnosis, confinement duration, economic impact, physical health effects of the pandemic, and adherence to confinement) and levels of depression. These findings suggest that people with higher levels of meaning-centered coping could maintain lower levels of depression, despite finding themselves in a similar situation as those with low levels of meaning-centered coping. A similar protective role of meaning-centered coping was observed in the relationship between some demographic vulnerability factors (age and economic status) and anxiety; and between gender and stress levels; and finally, between some risk factors (gender, economic status, physical health effects and lifestyle changes due to COVID-19) and general psychological distress (combined score of depression, stress, and anxiety).

The moderation effects of meaning-centered coping were most evident with regards to the depression subscale which is in line with previous studies reporting a close relationship between meaning in life and depression (e.g., Steger et al., 2006; Disabato et al., 2017; Carreno et al., 2020). Depression, as measured by the DASS-21, is characterized by meaninglessness in life, hopelessness, and lacking interest and initiative (i.e., low levels of positive affect; Lovibond and Lovibond, 1995). The MCCS, on the other hand, is defined by quite the opposite: meaning in life, maintaining hope, prosociality, and proactivity. One potential explanation of these findings is that meaning-centered coping is strongly related to the maintenance of positive affect in the face of adversity, and has both a diminishing and transformative power over negative affect. Overall, these findings suggest that while the intensity of the COVID-19-related conditions correlates with the degree of presence of psychological distress, this relationship is highly modulated by meaning-centered coping, diminishing their harmful effects, especially in the case of depressive symptoms. They provide additional evidence of the role of meaning in life in promoting resilience, as has been previously suggested (for a review see Wong, 2012; Hicks and Routledge, 2013; Batthyany and Russo-Netzer, 2014).

The above-mentioned findings may offer empirical support to existential positive psychology (e.g., Wong, 2009, 2011a) and highlight the importance of strategies and interventions targeting not only the reduction of negative affect but also the transformation of adversities into growth and increasing positive affect. These strategies are integrated in positive psychology (for interventions, see for instance, Craske et al., 2019; Carr et al., 2020; Hendriks et al., 2020) and especially in existential positive psychology (e.g., Wong, 2010, 2012, 2020a). Interventions based on these considerations may be extremely relevant during the COVID-19 crisis, as the experience of negative affect is unavoidable for a large part of the population.

Although the results show a potentially relevant role of meaning-centered coping in the COVID-19 era, there are some limitations we ought to discuss. First and foremost, since it is a cross-sectional study, causal directionality of the relationships here investigated cannot be confirmed. The relationship may even be the other way around, that is, lower levels of psychological distress may lead to more meaning-centered coping. Although the moderating effects of meaning-centered coping between vulnerability factors and psychological distress make our interpretation feasible, it is possible that the relationship may be circular: this type of coping leads to lower levels of psychological distress (i.e., more positive feelings) which in turn leads to more frequent use of adaptive coping strategies (for the role of positive emotions in broadening the individual’s thought-action repertoire, see Fredrickson, 2004). Future longitudinal studies should address this issue. Second, we did not use a representative sample. For instance, females were overrepresented which limits the generalizability of the findings. Third, we focused on general, cross-cultural trends although controlling for the effect of country. Future studies may look at similar phenomena independently in each country. Fourth, the cutoff point of the duration of confinement for a statistically significant effect was not assessed. This variable also reflects on the data collection period of each country during the rapidly changing situation (for instance, data in Spain was collected first, and data in the United States was collected last, hence the differences). These differences based on the data collection period were partially mitigated by the inclusion of the variable “severity index” that intended to add an objectively comparable score of the situation of the pandemic in each country during their respective data collection periods. Fifth, we used self-reported measures that can be biased. Future studies using behavioral and physiological measures could yield more robust results on the protective role of meaning-centered coping.

To conclude, the results of the present study point to a critical role of meaning-centered coping in attenuating the detrimental effects of the COVID-19 pandemic on psychological distress, especially on depressive symptoms. These effects were observed in a global sample, including more than 12,243 participants from all continents. This protective effect seems to be exerted via two different mechanisms: by diminishing the emergence of all types of psychological distress and acting as a moderator between hardship and such symptoms. The findings suggest that future interventions from an existential positive psychology point of view that foster meaning-centered coping can be beneficial, especially for members of vulnerable groups.

We urge decision-makers not to place the focus only on risk factors and strategies to ameliorate the magnitude of the COVID-19-related situations and make more emphasis on fostering psychological resilience and promoting a proactive attitude in individuals so that they do not regard themselves as passive sufferers of threats. Promoting effective coping mechanisms such as meaning-centered strategies may prove to be adequate in the current situation and prescriptions issued by institutions like the World Health Organization could benefit millions of people by integrating these considerations.

## Data Availability Statement

The raw data supporting the conclusions of this article will be made available by the authors, without undue reservation.

## Ethics Statement

The studies involving human participants were reviewed and approved by – Comisión de Bioética, Universidad de Almería – Ethics Commission for Research within the Faculty of Psychology and Educational Sciences, Universitatea “Alexandru Ioan Cuza” Din Iaşi – Comité de Ética en Investigación, Universidad de Monterrey – Ethics Commission Batna 1 University Rectorat – Department of Applied Psychology, Guru Jambheshwar University of Science and Technology – Comissão Nacional de Ética em Pesquisa – Medizinische Fakultät Ethikkommission, Heinrich-Heine-Universität Düsseldorf – Kutatásetikai Bizottság, Eötvös Loránd Tudományegyetem, Pedagógiai és Pszichológiai Kar – Ethics Commission, International University of Business Agriculture and Technology (IUBAT) – Comitato Etico Della Ricerca Psicologica (AREA 17) Dipartimenti/Sezione di Psicologia – Università di Padova – Institutional Review Board, Lebanese American University – Ethical Review Board COMSATS University Islamabad, Lahore Campus – Ethics Commission, Komisija za Etiko Filozofske Fakultete, Filozofska Fakulteta, Univerze v Ljubljani – Ethics Commission, Pathumwan Institute of Technology – Ethics Commission, Örebro Universitet – Institutional Review Board, Rutgers University – Faculty Ethics Committee, University of South Wales – Institutional Review Board, Ağri İbrahi̇m Çeçen Üni̇versi̇tesi̇ Rektörlüğü. The patients/participants provided their written informed consent to participate in this study.

## Author Contributions

NE contributed to the manuscript by coordinating the data collection, theoretical framing, text writing, and data analysis. JAP-E contributed to the manuscript by theoretical framing, text writing, and offering a critical revision of the manuscript. DC contributed to the manuscript by coordinating the data collection, theoretical framing, text writing, and revision of the manuscript. All authors contributed to the article and approved the submitted version.

## Conflict of Interest

The authors declare that the research was conducted in the absence of any commercial or financial relationships that could be construed as a potential conflict of interest.
