# Corrigendum: Meaning-Centered Coping in the Era of COVID-19: Direct and Moderating Effects on Depression, Anxiety, and Stress

**DOI:** 10.3389/fpsyg.2021.682447

**Published:** 2021-05-05

**Authors:** Nikolett Eisenbeck, David F. Carreno, José Antonio Pérez-Escobar

**Affiliations:** ^1^Department of Psychology, University of Almería, Almería, Spain; ^2^Department of Personality, Evaluation and Psychological Treatment, Faculty of Psychology, University of Seville, Seville, Spain; ^3^Chair of History and Philosophy of Mathematical Sciences, Department of Humanities, Social and Political Sciences, ETH Zürich, Zurich, Switzerland

**Keywords:** COVID-19, meaning-centered coping, stress appraisal, psychological distress, depression, anxiety, existential positive psychology, positive psychology (PP1.0 and PP2.0)

After discussing the character of the contributions to the final version of the paper, we have decided that the author order should be rearranged.

The corrected author list is shown below.

Nikolett Eisenbeck^1, 2*^, David F. Carreno^1^ and José Antonio Pérez-Escobar^3^

The authors apologize for this error and state that this does not change the scientific conclusions of the article in any way. The original article has been updated.

